# Supplementary material for: Evaluation of Novel Guanidino-Containing Isonipecotamide Inhibitors of Blood Coagulation Factors against SARS-CoV-2 Virus Infection
Source: Viruses. 2022 Aug 5;14(8):1730. doi: 10.3390/v14081730 (PMC9415951; doi:10.3390/v14081730)
Supplement: Supplementary file 1 [file viruses-14-01730-s001.zip › viruses-1782051-supplementary.pdf]

Research article

Evaluation of novel guanidino-containing isonipecotamide inhibitors of blood coagulation factors against SARS-CoV-2 virus infection

Flavio De Maio,[1,¶] Mariagrazia Rullo,[2,¶] Modesto de Candia,[2] Rosa Purgatorio,[2] Gianfranco Lopopolo,[2#] Giulia Santarelli,[1] Valentina Palmieri,[1] Massimiliano Papi,[1] Gabriella Elia,[3] Erica De Candia,[4] Maurizio Sanguinetti,[1,*] Cosimo Damiano Altomare[2]

[1]*Dipartimento di Scienze di Laboratorio e Infettivologiche, Fondazione Policlinico Universitario A. Gemelli IRCCS, Rome, Italy*

[2]*Department of Pharmacy–Pharmaceutical Sciences, University of Bari Aldo Moro, Bari, Italy*

[3]*Department of Veterinary Medicine, University of Bari Aldo Moro, Bari, Italy*

[4]*Department of Translational Medicine and Surgery, Catholic University of Rome, Rome, Italy*

* Corresponding author
E-mail address: maurizio.sanguinetti@unicatt.it (M. Sanguinetti)

¶ These authors equally contributed

# Present address: gianfranco.lopopolo@newchemspa.it; Research & Development Dept., Newchem SpA, via Roveggia 47, 37138 Verona

SUPPLEMENTARY DATA

1. Factor Xa and thrombin inhibition assays p. 2

2. Stability in aqueous buffer and human serum at pH 7.4 p. 3

3. Concentration-response relationships of SARS-CoV-2 infectivity after treatment with nafamostat mesylate and compound **3** p. 4

1. Factor Xa and thrombin inhibition assays

The time-dependent inhibition of factor Xa (fXa) and thrombin (thr) was assessed by determining the hydrolysis rates of the selective synthetic chromogenic substrates, which were monitored at 405 nm. Enzymes and substrates used were (final concentrations): 0.41 unit·ml^{-1} for bovine thrombin (Sigma−Aldrich, Milan, Italy), and 50 μM S-2238 (D-Phe-Pip-Arg-*p*-NA) from Chromogenix AB-Instrumentation Laboratories (Milan, Italy); 4 nM recombinant human factor Xa and 0.04 μM S-2765 (Z-D-Arg-Gly-Arg-p-NA) from Chromogenix AB-Instrumentation Laboratories (Milan, Italy).

The enzyme solutions (100 μl) were mixed with 2 μl of DMSO solution containing the test compound or DMSO alone as control, and incubated at different times (5, 15 and 30 min). Reactions were initiated by adding 100 μl of substrate solutions, and the increase in absorbance was monitored for 10 minutes. The initial velocities were determined, and the concentrations of the inhibitors required to reduce the control velocity by 50% (IC_{50}) were determined by a sigmoidal regression. Each kinetic was repeated three times. After determination of the IC_{50} values, the inhibition constants (K_i) were calculated by applying the Cheng−Prousoff equation (Tables S1A-B).

Table S1A. Constant (K_i) values of time-dependent inhibition, after co-incubation of thrombin with nafamostat mesylate (NAF) and compound **3**.

	**K_i (nM), thrombin[a]**		
**Incubation time**	**0 min**	**15 min**	**30 min**
**NAF**	77 ± 7	13 ± 9	5 ± 4
**3**	15960 ± 40	16700 ± 17	15000 ± 11

Table S1B. K_i values of time-dependent inhibition, after co-incubation of fXa with nafamostat mesylate (NAF), and compound **3**.

	**K_i (nM), factor Xa[a]**		
**Incubation time**	**0 min**	**15 min**	**30 min**
**NAF**	363 ± 6	340 ± 10	225 ± 9
**3**	20 ± 7	17 ± 8	15 ± 5

[a]K_i values determined by applying the Cheng–Prousoff equation to IC_{50} values; regression analysis was carried out using GraphPad Prism software ver. 5.01. Values are expressed as mean ± SD; data are means of three independent measurements, each one performed in triplicate.

2. Stability in aqueous buffer and human serum at pH 7.4

Table S2. Time dependent residual, as % of the starting concentration, of nafamostat mesylate (NAF) and compound **3** in buffered aqueous solution (pH 7.4) and in pooled human serum.

**Time (h)**	**NAF**		**Cmpd 3**	
	pH 7.4 buffer	*human serum*	*pH 7.4 buffer*	*human serum*
0	100	100	100	100
0.5	95.1±0.5	45.9±4.3		
1	87.6±1.2	21.2±5.2	98.8±0.5	95.8±1.0
2	84.9±1.0	5.1±3.3		
4	81.6±1.8		98.1±1.5	96.6±1.5
8	78.4±2.5			
12	73.8±3.0		97.5±0.5	93.2±2.5
24	61.4±2.8		97.9±1.0	96.8±3.0

3. Concentration-response relationships of SARS-CoV-2 infectivity of nafamostat mesylate and compound 3

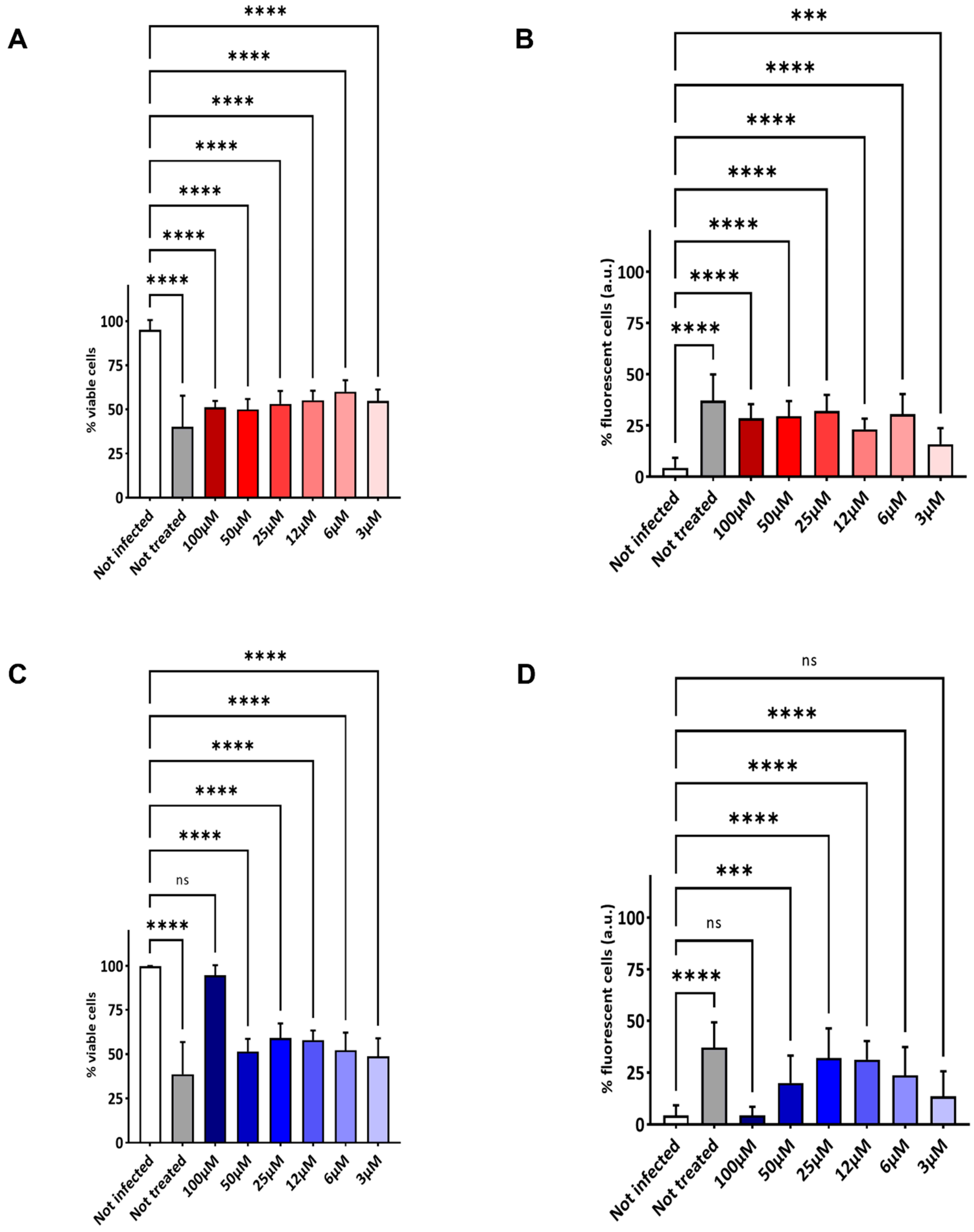

Figure S1. Compound 3, and not nafamostat mesylate (NAF), protects from SARS-CoV-2 infection at the highest concentration tested (100 µ). VERO cells were pre-treated with nafamostat mesylate and compound **3** at different concentrations (from 100 µM to 3 µM) for 2 hours before being infected with SARS-CoV-2. Two hours later, infection solution was

removed, and new fresh sterile medium was added. Cells were monitored daily until monolayer was fixed before staining with crystal violet (Panel A and C, showing the effects of NAF and **3**, respectively) or carrying out immunofluorescence assay (Panel B and D for NAF and **3**), to evaluate cytopathic effect and the intracellular viral load, respectively. Cytation was used to acquire images of each well and analysis was performed by ImageJ software measuring the integrity of the cell monolayer. Bar plots, showing average effect and standard deviation, summarize the results of repeated experiments. Data were analyzed by using Nested one-way ANOVA comparison test followed by Dunnett's correction. Statistically significant results are indicated according to the *p* value as follows: * $p < 0.05$; ** $p < 0.01$; *** $p < 0.001$; **** $p < 0.0001$ (each sample was compared with the not infected control).
